# Physicians’ views and knowledge on the antibiotic treatment of pneumonia in advanced dementia

**DOI:** 10.1186/s13584-024-00649-1

**Published:** 2024-12-16

**Authors:** Itai Harpaz, Boris Punchik, Tamar Freud, Yan Press

**Affiliations:** 1 Joyce & Irving Goldman Medical School, Faculty of Health Sciences, Ben-Gurion University of the Negev, Beer-Sheva, Israel; 2 Clalit Health Services, Community Division, Ramat-Chen Brull Mental Health Center, Tel Aviv-Yafo, Israel; 3 Faculty of Health Sciences, Ben-Gurion University of the Negev, Beer- Sheva, Israel; 4 Unit for Community Geriatrics, Division of Health in the Community, Ben-Gurion University of the Negev, Beer-Sheva, Israel; 5 Home Care Unit, Clalit Health Services, Beer-Sheva, Israel; 6 Siaal Research Center for Family Medicine and Primary Care, Faculty of Health Sciences, Ben- Gurion University of the Negev, Beer- Sheva, Israel; 7 Department of Geriatrics, Soroka Medical Center, Beer-Sheva, Israel; 8 Center for Multidisciplinary Research in Aging, Ben-Gurion University of the Negev, Beer- Sheva, Israel; 9 Geriatric Department, Unit for Community Geriatrics, Division of Health in the Community, Soroka Medical Center, Ben-Gurion University of the Negev, Beer-Sheva, Israel

**Keywords:** Physicians, Pneumonia, Advance dementia, Antibiotic treatment

## Abstract

**Background:**

Antibiotic treatment (AT) for patients with advanced dementia and pneumonia is a complex issue. AT can prolong life, but it can also prolong suffering for the patient and the family. In this study we evaluated physicians’ attitudes to this issue.

**Methods:**

A vignette-based survey among community-based physicians (CBP) and hospital-based physicians (HBP) who work in southern Israel. The physicians were asked to complete a questionnaire on their knowledge and attitudes to AT, based on a case description of a patient with advanced dementia and pneumonia.

**Results:**

211 physicians participated in the study including 134 CBP (63.5%) and 77 HBP (36.5%). 177 physicians chose the AT option for the patient presented in the case, with 59.7% of the HBP and 32.8% of the CBP choosing intravenous (IV) AT (*p* = 0.017). In contrast, in the same case, but with themselves as the patient, 68.8% of HBP and 32.8% of CBP chose the option of palliative care without AT, with only 14.3% of HBP and 10.4% of CBP choosing the option of hospitalization with IV AT. In two logistic regression models, physicians who chose IV AT for themselves were fourfold more likely to make a similar choice for their patients. Over 75% of the physicians were not knowledgeable about the administration of AT in accordance with the Dying Patient Act.

**Conclusions:**

The results of this study indicate the need for an educational intervention among physicians to expand their knowledge and expertise on end-of-life treatment for patients with advanced dementia. In addition, we invite policy makers to convene a discussion on the possibility of changing the law to facilitate the conduct of studies of patients with advanced dementia. Such studies would make it possible to develop an evidence-based treatment strategy.

## Background

Dementia is a progressive disease that eventually causes dependence and death [1]. In its advanced stages dementia is a terminal disease with life expectancy like metastatic breast cancer and stage 4 heart failure, at an average of 1.3 years [2]. Patients with advanced dementia are at high risk of developing pneumonia [3,4], with a reported mortality rate resulting from pneumonia above 40% ^[3, 5, 6^.

The rate of use of antibiotic treatment (AT) in patients with advanced dementia and pneumonia varies among countries [7,8] and increases with time [5].

The literature on the contribution of AT to improved survival of patients with advanced dementia and pneumonia is not conclusive. Some studies reported positive results [7,9], while in others no association was found between AT and improved survival [7,10,11]. Szafara et al. found that hydration rather than AT per se improved the prognosis of AT patients with pneumonia [12]. In any event, in studies that did find an association between AT and survival, there was no difference in the means of AT administration, i.e., oral, intramuscular (IM) or intravenous (IV) [8,9].

The literature is also inconclusive in relation to whether AT improves quality of life and prevents suffering. In some studies AT was found to improve the comfort of patients with advanced dementia and pneumonia [6,13], while in other studies the association was inverse [9,14]. This lack of consistency can be explained, at least in part, by the possibility that the nursing staff is focused on AT administration and invests less time in personal contact with patients and their level of comfort [15]. Furthermore, invasive therapy and, in some cases, hospitalization can increase the suffering of patients [16].

Israel has a regulation policy for the administration of AT in end-of-life patients. Under the “Dying Patient Act 2005”, Israel’s dying patient law, choosing palliative care alone without administering AT to a person with pneumonia and advanced dementia is defined as avoiding “ancillary care.” According to the law, avoidance of ancillary care is permitted only when a patient is in the “final stage of the disease” and “his life expectancy, even if he/she is given medical treatment, does not exceed two weeks” [17]. According to the law, physicians in Israel *must* administer AT to a patient with pneumonia and advanced dementia until the patient reaches the last two weeks of life.

It is important to note that the Israeli law does not instruct physicians as to the mode of AT administration (oral, IM, or IV). To our knowledge there are no data from Israel on the use of AT in the last two weeks of life for patients with advanced dementia.

Furthermore, in most cases it is difficult to assess precisely whether the patient is in their last two weeks of life or not, so the decision on AT would appear to be based on advanced medical directives, the opinion of the primary caregiver and the family, and to a great extent the physician’s discretion.

The aim of the present study was to evaluate the knowledge and attitudes of physicians working in southern Israel on the administration of AT to patients with advanced dementia and pneumonia.

## Methods

This study was a vignette-based survey among two populations of physicians.

### Study population

The first group was community-based physicians (CBP), physicians who work in primary care clinics in the southern region of the Clalit Healthcare Services. The physicians in those clinics are family physicians, specialists and residents, and general practitioners.

The second group was hospital-based physicians (HBP), who worked in internal medicine wards and the emergency room of the Soroka University Medical Center, the largest tertiary care center in southern Israel. This group included board-certified internists, residents in internal medicine, family physicians on a hospital rotation, and board-certified physicians and residents in emergency care medicine.

During their training, family physicians, in the first year of their training, participate in a structured course on palliative medicine for several days and are required to carry out on-call duties in a home hospice unit. To our knowledge internists and emergency medicine physicians do not receive any formal training in palliative medicine.

### Study tool

The study tool was a self-administered questionnaire, in which the participants were presented with the following case:

Samuel, 89, a widower and father of a daughter (the primary caregiver), who has been living at home with a migrant worker, was diagnosed with Alzheimer’s disease 10 years earlier. In the last 48 h he developed a fever (38.3 ℃) and a productive cough. On auscultation, crepitations were heard in the lower right lobe and the respiration rate was 28 breaths per minute. In the past six months he frequently coughed while eating and had difficulty swallowing, suffered from severe memory impairment, did not recognize his daughter, was bedridden, could mutter syllables only, was unable to perform any basic activities such as mobility, bathing, dressing, eating/drinking, or sphincter control. Samuel had no advance directives or a legally appointed guardian. His daughter asked that Samuel not suffer.

The questionnaire included questions on the knowledge and attitudes of physicians regarding the treatment of pneumonia in patients with advanced dementia, as well as professional characteristics: primary workplace, professional status, specialization, years of seniority, country of medical studies, and socio-demographic characteristics including age, gender, and country of birth.

In addition, the physicians were asked how they would want to be treated if they themselves were in a situation like that described in the case.

The questionnaires were handed out to physicians during staff meetings in wards and clinics. Physicians who agreed to participate in the study filled out the questionnaire.

### Sample size

The sample size to prove the study hypothesis with 80% power, and 95% probability was 203 physicians: 122 CBPs and 81 HBPs. The calculation was based on the results of a previous study that examined physicians’ attitudes, in which 60% of the family physicians agreed to help advanced dementia patients end their lives, compared with 39% of physicians from other specialties [18]. Furthermore, the sample size calculation was weighted to account for the fact that there are 1.5 times more physicians working in the community than in the hospital.

### Data analysis

Data analyses was performed using the IBM SPSS 29 statistic software.

Univariate analyses comparing physicians’ views and knowledge regarding the treatment of pneumonia in advanced dementia between the two study groups (CBP vs. HBP) were performed using Chi-square test for categorical variables and t-tests for continuous variables.

Univariate analyses were also conducted to compare physicians’ characteristics according to their preferred treatment option for pneumonia in advanced dementia patients: (a) hospitalize the patient and give him/her IV AT, (b) administer IM AT without hospitalization, (c) provide oral AT without hospitalization, or (d) no AT, palliative treatment only.

A multivariate logistic regression model was built to predict physicians who would choose hospitalization and IV antibiotic therapy as the treatment for their patient.

Statistical significance was set at *p* < 0.05 for all tests.

The Helsinki Committee of the Soroka Medical Center granted the study an exemption from the need for informed consent.

## Results

### Participants’ characteristics

In total, 211 doctors completed the study questionnaire: 77 HBP (36.5%), and 134 CBP (63.5%). The mean age was 41.6 ± 10 years, with 88 (41.7%) females and 123 (58.3%) males. The mean seniority was 15 ± 10.9 years. Seventy physicians (33.2%) were board-certified in family medicine, 39 (18.5%) in internal medicine, and 6 (2.8%) in emergency medicine. Ninety-one physicians (43.1%) were residents, and 5 (2.3%) were general practitioners without specialization.

A comparison of sociodemographic characteristics between the two study groups is presented in Table 1. HBPs were older and more experienced, CBPs were more likely to have been born and studied medicine abroad. In addition, the two study groups practiced different religions, but had similar degrees of religiosity, similar representation of both genders, and equal representation of board-certified doctors.

**Table 1:** Comparison of sociodemographic characteristics between HBP and CBP

	HBP (*N* = 77)	CBP (*N* = 134)	*p*-value
**Age (years)**,** mean ± SD**	43.7 ± 8.9	38.1 ± 10.9	< 0.001
**Gender (male)**,** n (%)**	51 (66.2)	72 (53.7)	0.083
**Religion**,** n (%)**			
Judaism	48 (62.3)	80 (59.7)	0.005
Islam	57 (35.1)	32 (23.9)	
Other	2 (2.6)	22 (16.4)	
**Degree of religiosity**,** n (%)**			
Secular	39 (50.6)	82 (61.2)	0.179
Religious /Traditional	38 (49.3)	52 (38.8)	
**Country of birth (Israel)**,** n (%)**	58 (75.3)	52 (38.8)	< 0.001
**Country of medical studies (Israel)**,** n (%)**	37 (48.1)	28 (20.9)	< 0.001
**Professional seniority (years)**,** mean ± SD**	17.2 ± 9.8	11.4 ± 11.7	< 0.001
**Professional Status**,** n (%)**			
Specialist	39 (50.6)	79 (56.7)	0.117
Resident	38 (49.4)	53 (39.6)	
General Practitioner	0 (0.0)	5 (3.7)	
**Specialization**,** n (%)**			
Internal medicine	66 (85.7)	6 (4.5)	< 0.001
Family medicine	1 (1.3)	128 (95.5)	
Emergency medicine	10 (13.0)	0 (0.0)	

HBP-Hospital-based physicians; CBP-Community-based physicians

### Physicians’ attitudes and knowledge regarding the treatment of pneumonia in advanced dementia

The preferred treatment of pneumonia in patients with advanced dementia for most physicians was AT (any mode of administration) (*N* = 177, 83.9%). Only 34 (16.1%) chose palliative care without AT. A comparison of the knowledge and attitudes between the two groups is presented in Table 2. There were differences between HBP and CBP in the preferred mode of AT (*P* = 0.005). When the treatment options were condensed into two groups, hospitalization with IV AT vs. other options, HBP tended to choose the more invasive treatment (59.7% vs. 42.5%, *P* = 0.02). 20.8% of HBP and 13.4% of CBP (*P* = 0.177) chose palliative care without AT. 96.1% of HBP vs. 80.6% of CBP chose the preservation of quality of life and patient comfort as a primary goal (*P* = 0.001).

**Table 2:** Comparison of physicians’ attitudes and knowledge regarding the treatment of pneumonia in advanced dementia, between the two study groups

	HBP (*N* = 77)	CBP (*N* = 134)	*p*-value
**Chosen treatment for a patient with pneumonia and advanced dementia**,** n (%)**			
Hospitalization and IV antibiotic treatment	46 (59.7)	57 (42.5)	0.005
IM antibiotic treatment without hospitalization	6 (7.8)	25 (18.7)	
PO antibiotic treatment without hospitalization	9 (11.7)	34 (25.4)	
No antibiotic treatment, palliative care only	16 (20.8)	18 (13.4)	
**Chosen treatment for a patient with pneumonia and advanced dementia (grouped answers)**,** n (%)**			
Hospitalization and IV antibiotic treatment	46 (59.7)	57 (42.5)	0.017
Other	31 (40.3)	77 (57.5)	
**The purpose of the treatment for pneumonia in advanced dementia**,** n (%)**			
Prolongation of life	3 (3.9)	26 (19.4)	0.001
Maintaining quality of life and comfort	74 (96.1)	108 (80.6)	
**Is the physician familiar with the “Dying Patient Act**,** 2005”?**,** n (%)**			
Familiar	19 (24.7)	32 (23.9)	0.999
Not familiar	58 (75.3)	102 (76.1)	
**The level of involvement of the primary caregiver**,** n (%)**			
The primary caregiver makes the decision, I only present the options and their meaning	10 (13.0)	23 (17.2)	0.538
The primary caregiver is a participant in treatment selection together with me	39 (50.6)	71 (53.0)	
I will involve the primary caregiver in the meaning of the treatment, but choose the treatment myself	28 (36.4)	40 (29.9)	
**Self-estimated number of patients with pneumonia and advanced dementia that were treated by the physician in the past year**,** mean ± SD**	54.1 ± 70.1	3.7 ± 4.5	< 0.001
**Treatment preferences**,** if the doctor himself/herself would suffer from advanced dementia and pneumonia**,** n (%)**			
No antibiotic treatment, palliative care only	53 (68.8)	44 (32.8)	< 0.0001
PO or IM antibiotic treatment without hospitalization	13 (16.9)	76 (56.7)	
Hospitalization and IV antibiotic treatment	11 (14.3)	14 (10.4)	
**Is the treatment preference for myself the same as the treatment I chose for the patient?**,** n (%)**			
Treat differently	47 (61.0)	80 (59.7)	0.885
Treat the same	30 (39.0)	54 (40.3)	

HBP-Hospital-based physicians; CBP-Community-based physicians

Only 24.7% of HBP and 23.9% of CBP (*P* = 0.999) showed “sufficient knowledge” about AT, based on the “Dying Patient Act 2005,” by answering the question correctly: “It is allowed to treat a dying patient with palliative care alone, without antibiotics, if the patient’s life expectancy is shorter than 2 weeks.” Only 13% of HBP and 17.2% of CBP agreed to base their decision solely on the will of family members (*P* = 0.538).

In response to the question as to their personal treatment preferences if they themselves were in the situation of the patient in the vignette, there was a significant difference between the physician groups in which 68.8%of HBP and 32.8% of CBP chose the option of palliative care without AT, 16.9% of HBP and 56.7% of CBP chose oral or IM AT without hospitalization, and 14.3% of HBP and 10.4% of CBP preferred the option of hospitalization with IV AT (*P* < 0.001).

No difference was found between HBPs and CBPs in their approach to the level of involvement of primary caregivers in making decisions about AT (*p* = 0.538). While most physicians in both groups considered the primary caregiver a partner in decision-making, it is important to note that 36.4% of HBPs and 29.9% of CBPs responded that they would consider the primary caregiver’s opinion, but the final decision would be theirs. The self-estimated number of patients with pneumonia in advanced dementia that were treated by a physician in the past year was higher among HBPs than CBPs (54.1 ± 70.1 vs. 3.7 ± 4.5, *p* < 0.001).

Only 39% of HBP and 40.3% of the CBP chose the same treatment option for their patients and for themselves if they were in the same position (*P* = 0.885).

### Treatment options for pneumonia in advanced dementia patients

Table 3 shows the results of the univariate analysis on physician characteristics and their choice of more intensive treatment for pneumonia (hospitalization and IV AT). The physicians who chose the more intensive treatment option were younger (40.1 ± 10.7 vs. 43.1 ± 9.2 years, respectively, *P* = 0.005), with lower work seniority (14.0 ± 11.3 vs. 16.1 ± 10.5 years, respectively, *P* = 0.037). CBP comprised 55.3% of the physicians who chose the more intensive treatment option and were 71.3% of those who chose the less intensive option (*P* = 0.024). Physicians who chose the more intensive treatment option for themselves also chose it for their patients (18.5% vs. 5.6%, respectively, *P* = 0.007).

**Table 3:** Comparison of physicians’ characteristics according to their preference between two treatment options for pneumonia in advanced dementia patients

	Hospitalization and IV AT (*N* = 103)	Other options (*N* = 108)*	*p*-value
Age, years, mean ± SD	40.1 ± 10.7	43.1 ± 9.2	0.005
Gender (male), n(%)	60 (58.3)	63 (58.3)	1.0
Religion (Judaism), n(%)	58 (56.3)	70 (64.8)	0.261
Degree of religiosity (Secular), n(%)	55 (53.4)	67 (62.0)	0.258
Country of birth (Israel), n(%)	53 (51.5)	57 (52.8)	0.957
Country of MD graduation (Israel), n(%)	29 (28.2)	36 (33.3)	0.506
Professional seniority, years, mean ± SD	14.0 ± 11.3	16.1 ± 10.5	0.037
Professional status (specialist), n(%)	53 (51.5)	62 (57.4)	0.466
Specialization (Family Medicine), n(%)	58 (56.3)	71 (65.7)	0.206
Main workplace (Community), n(%)	57 (55.3)	77 (71.3)	0.024
Familiar with “Dying Patient Act, 2005”, n(%)	20 (19.4)	31 (28.7)	0.157
The purpose of the treatment for pneumonia in advanced dementia is maintaining quality of life and comfort (yes), n(%)	85 (82.5)	97 (89.8)	0.181
Self-estimated number of patients with pneumonia in advanced dementia that were treated by the physician in the past year, mean ± SD	23.8 ± 46.7	20.6 ± 51.0	0.139
Self-treatment preference if the physician himself/herself had pneumonia and advanced dementia is hospitalization and IV antibiotic therapy, n(%)	19 (18.5)	6 (5.6)	0.007

IV- intravenous; AT- antibiotic treatment

*Other options: IM AT without hospitalization, Oral AT without hospitalization, no antibiotic treatment, palliative care only

Two logistic regression models (Table 4) were developed to predict physicians’ preference for hospitalization and IV AT. In both models, variables that reached statistical significance in the univariate analyses were included and were adjusted for the physician’s age. Since there is a correlation between age and physician seniority these variables were entered into separate models.

**Table 4:** Multivariate logistic regression models to predict physicians who would choose hospitalization and IV antibiotic therapy as a treatment for pneumonia in advanced dementia patients

Model	Variable	OR	95% CI		P value	R square
			Lower	Upper		
1	Age (years)	1.024	0.994	1.054	0.121	0.105
	Gender (Male)	1.384	0.769	2.493	0.278	
	Work place (Community)	0.554	0.301	1.019	0.057	
	Treatment preference if the doctor himself/herself had pneumonia and advanced dementia is hospitalization and IV (Yes)	4.132	1.526	11.188	0.005	
2	Professional seniority (years)	1.011	0.985	1.039	0.403	0.095
	Gender (Male)	1.367	0.760	2.461	0.297	
	Work place (Community)	0.523	0.285	0.957	0.036	
	Treatment preferences if the doctor himself/herself had pneumonia and advanced dementia is hospitalization and IV (yes)	4.107	1.520	11.097	0.005	

In the first model, which was adjusted for age, the physician’s personal preference for hospitalization and IV AT, increased the chance that the patient would also get this treatment. This was the only variable that reached statistical significance (OR = 4.132, 95% CI1.526-11.188, *P* = 0,005). In the second model the same variable increased fourfold the chance of recommending hospitalization and IV AT for the patient (OR = 4.107, 95% CI 1.520-11.097, *P* = 0.005). Being a CBP reduced the chance of recommending this treatment for patients (OR = 0.523, 95% CI 0.285–0.957, *P* = 0.036). It should be noted that both the models were weak (R [2] 0.105, 0.095).

## Discussion

In the present study, 117 of 211 physicians (83.9%) chose to treat the patient in the case vignette with some form of AT. The question of treatment preference has been evaluated in several studies in the past and a search of the literature revealed differences on this subject among different countries. For example, among 288 physicians in Italy [19], 14% chose the option without AT when the patient’s life expectancy was less than six months, and 36% chose it when the life expectancy was less than one month. Since in the present vignette life expectancy did not exceed six months, one can conclude that there was no difference between the physicians in the Italian study and ours. In contrast, in a study of physicians in Brazil [20] only 55% of the physicians chose to prescribe AT in similar cases. In a study of Canadian physicians who were presented with the case of a patient who had previously refused to write advance directives and now suffered from advanced dementia and a life-threatening infection, only 2% of the physicians chose AT [18]. In the present study, which compared HBP and CBP, there was no difference between the groups in the rate of preference for palliative care without AT. In contrast, a study from France [21] that was conducted among physicians treating terminal patients in the community and in various hospital settings found that community physicians were less likely to give AT (56%) compared to physicians who worked in other settings (78%). The authors explain this, among other reasons, by significant differences in the number of terminal patients treated by community physicians compared to physicians in hospital settings. In the present study we found similar differences between HBP and CBP in the number of such patients that they treated, but this did not affect the physician’s decision to prefer palliative care alone.

The finding that the self-estimated number of patients with pneumonia and advanced dementia treated by CBPs in our study was very low (Table 2) deserves a separate discussion. As noted previously, community physicians in the study by Durand et al. [21] treated a similar number of palliative patients. From our knowledge of the situation in Israel, most patients with advanced dementia are treated either in specialized units or in long-term care institutions, hence CBPs have less experience in managing these patients. Conversely, we believe that most patients with advanced dementia, when they develop pneumonia, are referred to hospitals, and this likely explains the greater experience of HBPs in caring for these patients. It is possible that more extensive experience with these patients could have altered the approach of CBPs towards AT (both for their patients and themselves).

It’s possible that differences in the number of patients under the care of physicians from the two research groups in the past year could account for variances found in responses to other questions. Compared to CBP, HBP preferred hospitalization with IV AT, saw the goals of preserving quality of life and preventing suffering (compared to extending life) as a primary aim of treatment for pneumonia, and more HBP chose palliative treatment without AT if they themselves were in a similar situation to that in the vignette.

Most physicians, in both groups, viewed the primary caregiver as a partner in decision-making on treatment options, yet about one-third of the physicians said that they would consider the primary caregiver’s opinion but make the final decision themselves (Table 2). In Israel, primary caregivers who are not the patient’s legal guardian do not have legal standing to make decisions. However, the willingness of some physicians to consider reaching decisions contrary to the opinion of the primary caregiver is of concern, providing additional justification for the need to enhance the process for advanced medical directives, and/or to appoint a legal guardian.

When we compared the characteristics of physicians who chose the more intensive option for patients (hospitalization with IV AT) with those who preferred other options, we found that of all the variables that were statistically significant in the univariable analyses (age, work seniority, place of work, and preference for self-treatment for themselves) only the preference for more intensive treatment for themselves was a statistically significant predictor in both logistic regression models. This finding seems to be logical (“what is good for me is good for my patient”), however it is not consistent with another finding that although 34 (16.1%) of all physicians chose only palliative care without AT for their patients, 97 (46.0%) preferred this option for themselves.

The finding that almost half of the physicians in this study preferred palliative care for themselves is not new. For example, in a study of physicians and nurses from the United States [22], two thirds of the participants did not want AT if they themselves had sustained significant brain damage without ability to communicate, and had pneumonia.

There are previous reports of differences in the approach of physicians between treatment for their patients and for themselves. In a previous study from Israel on HBP and CBP in southern Israel [23], that addressed the issue of feeding tube in a patient with advanced dementia, although a high proportion of physicians thought feeding tube prevents aspiration, pneumonia, and pressure sores, over two thirds responded that they would not be interested in getting feeding tube for themselves if they had advanced dementia and a feeding disorder. In another study [24] the authors also found similar differences in end-of-life preferences in which physicians chose palliative care for themselves more than they did for their patients. The author used the term “compassionate behavior” for this phenomenon, which was associated with physicians’ inability to practice “rules as to what is best for their patient” on themselves. One possible explanation for this phenomenon is that the decisions that physicians reach for patients depend, to a degree, on accepted societal norms, on the healthcare system, and on the specific place of work, as well as the influence of the patient’s family. When physicians decide on treatment for themselves, they feel liberated from these pressures and can go against the stream.

Another finding in the present study, which could be a source of concern, is the lack of expertise of physicians on the Dying Patient Act. Less than a quarter of the physicians, in both groups, knew that the law allows physicians to avoid AT treatment only in the patient’s last two weeks of life. Considering that most patients in Israel do not have advanced directives and the finding that 68 physicians (32.3%), in the present study, thought that they had to reach a treatment decision on their own, some of the patients might get treatment contrary to the law and to their wishes, and without family involvement. This highlights the need for a training process for physicians in the clinical and medicolegal aspects of end-of-life treatment. We found that CBP, most of them family physicians, despite having received theoretical and practical training in home hospice care, did not differ from HBP in knowledge of the Dying Patient Act in relation to AT. This brings up the need to revise the training curriculum in palliative medicine.

In a paper that presented the development of good clinical practice recommendations for AT at the end of life, Seaton et al. [25] noted that these recommendations should focus on three principal topics: partnership in future-related decision making, agreement on the goals and limits of treatment, and an ongoing monitoring of any AT that the patient receives. These recommendations justify the need for urgent action to increase awareness of advanced directives in the general population in Israel.

The results of this study raise another important issue related to the requirements of the Dying Patient Act 2005 concerning patients with advanced dementia. The literature search presented in this paper points to the absence of randomized controlled trials (RCT) in this area. The law’s directives are based in principle on Jewish tradition and the ideal of sanctity of life. However, in the absence of clear research proof, several questions of concern remain. First, when we prescribe AT for patients with advanced dementia, are we actually extending life? Second, we are not sure whether we are “adding years to life or life to years”, i.e., are we simply extending life at the expense of the quality of life of the patient and the patient’s caregiving family? In this situation it is it is unclear whether *primum non nocere* is to provide treatment or avoid it. Without doubt, an RCT would add to our understanding, but that is not possible considering the laws’ definitions and, and even if the law permitted such research, recruiting participants for such a study would pose significant challenges. For example, most patients with advanced dementia do not have a legal guardian and the appointment of one for an RCT is very problematic. We believe that the possible solution to this problem is an amendment to the patient’s advanced medical directives that will enable patients to participate in RCTs in the future if they reach a stage of advanced dementia. Such an amendment would make it possible to plan future studies with the help of a list of “potential participants.” If it would be possible at the time that advanced medical directives are prepared to sign consent for participation in a future study, it would be possible to conduct studies not only on AT but on other issues such as feeding tubes, hydration, et al.

Another important issue relates to the finding that physicians who participated in the study were more “liberal” towards themselves in terms of choosing AT for the described vignette. Primary care physicians should inform patients about different possibilities of care in certain situations but should not present their own preferences as a benchmark for personal decision-making. However, if a patient is interested in knowing what the physician would do if they were in a similar situation, it might be appropriate for the physician to share their personal perspective. After all, it may be true that “what is good for the physician is good for the patient.” This would be even more relevant if the law were to be amended to allow patients the option to not take AT for a longer period than the last two weeks of life.

The present study has several limitations. It was conducted among physicians who work in the community and in one hospital in southern Israel. It entailed a convenience sample of physicians who attended staff meetings, so we cannot be sure that the sample represents all the physicians who work in these settings. It is even less clear if the results can be generalized to physicians working in other regions of Israel, or physicians in other countries. The analysis of physicians’ attitudes was based on responses to self-administered questions and on direct observation of the physicians’ actual practice. Thus, it is possible that the study findings do not reflect the treatment approach of all the physicians for patients in real-life situations. We also based our estimate of the number of patients with pneumonia and advanced dementia treated during last year on physician self-report, so the actual number could be substantially different. Another significant limitation is that the physician’s expertise on the Dying Patient Act was based on only one, albeit important, question, so the results may not reflect the actual level of expertise of the physicians on other aspects of this law. Another inherent limitation is the study design: as this is a cross-sectional study without any intervention, we cannot put forward a proven training program.

The study has several strengths. To our knowledge this is the first study in Israel that evaluated the knowledge and attitudes of physicians on the issue of treatment for patients with advanced dementia and pneumonia. A broad range of physicians participated in the study including CBP and HBP, and residents and board-certified physicians, who belong to different religions and have varying degrees of religiosity.

## Conclusions

In summary, both CBP and HBP exhibited lack of expertise on the Dying Patient Act on one hand, and inconsistent approaches to treatment for patients with advanced dementia and pneumonia, on the other.

The study findings highlight the need to increase the level of awareness for advanced directives in this population and to improve physicians’ knowledge on end-of-life decisions.

In addition, we invite policymakers to initiate a discussion aimed at identifying legal and ethical ways to determine the possibility of participation in future studies on advanced dementia; not only on AT but on other issues such as feeding tubes, hydration, et al.

Such studies would make it possible to develop an evidence-based treatment strategy.

It is important to understand that the decision to provide antibiotics to patients with advanced dementia (IV or other) is still supported by most physicians, and this should be the starting point for further investigation and policy decision-making.

## Data Availability

Data may be made available upon reasonable request to the corresponding author.

## Abbreviations

AT: Antibiotic treatment
HBP: HOSPITAL-based physicians
CBP: Community-based physicians
IM: Intramuscular
IV: Intravenous
